# Mechanomodulation: Physical Treatment Modalities Employ Mechanotransduction to Improve Scarring

**DOI:** 10.3390/ebj3020021

**Published:** 2022-03-26

**Authors:** Ulrike Van Daele, Jill Meirte, Mieke Anthonissen, Tine Vanhullebusch, Koen Maertens, Lot Demuynck, Peter Moortgat

**Affiliations:** 1 OSCARE, Organisation for Burns, Scar Aftercare and Research, 2170 Antwerp, Belgium; jill.meirte@uantwerpen.be (J.M.); mieke.anthonissen@oscare.be (M.A.); koen.maertens@oscare.be (K.M.); peter.moortgat@oscare.be (P.M.); 2 Research Group MOVANT (Movement Antwerp), Department of Rehabilitation Sciences and Physiotherapy, University of Antwerp, 2000 Antwerp, Belgium; tine.vanhullebusch@uantwerpen.be (T.V.); lot.demuynck@uantwerpen.be (L.D.); 3 Department of Rehabilitation Sciences, KU Leuven, 3001 Leuven, Belgium; 4 Department of Clinical and Lifespan Psychology, Vrije Universiteit Brussel, 1040 Brussels, Belgium

**Keywords:** scars, remodeling, mechanotransduction, mechanomodulation, physical scar management, dose dependency

## Abstract

Every year, surgical interventions, traumatic wounds, and burn injuries lead to over 80 million scars. These scars often lead to compromised skin function and can result in devastating disfigurement, permanent functional loss, psychosocial problems, and growth retardation. Today, a wide variety of nonsurgical scar management options exist, with only few of them being substantiated by evidence. The working mechanisms of physical anti-scarring modalities remained unclear for many years. Recent evidence underpinned the important role of mechanical forces in scar remodeling, especially the balance between matrix stiffness and cytoskeleton pre-stress. This perspective article aims to translate research findings at the cellular and molecular levels into working mechanisms of physical anti-scarring interventions. Mechanomodulation of scars applied with the right amplitude, frequency, and duration induces ECM remodeling and restores the ‘tensile’ homeostasis. Depending on the scar characteristics, specific (combinations of) non-invasive physical scar treatments are possible. Future studies should be aimed at investigating the dose-dependent effects of physical scar management to define proper guidelines for these interventions.

## 1. Introduction

Every year, over 80 million scars are produced in the developed world during surgical procedures or caused by traumatic or burn wounds [1]. Scars often lead to compromised skin function and can result in devastating disfigurement, permanent functional loss, psychosocial problems, and growth retardation [2,3,4,5]. Consequently, sequelae from scarring pose a significant burden, and the development of novel treatments is of paramount importance [6].

Unfortunately, to date, few substantially efficacious, nonsurgical, therapeutic scar management options exist. Furthermore, the options that do exist typically lead to only modest improvements after multiple treatment sessions [7,8,9,10,11].

Recent studies highlighted the importance of mechanical forces in wound healing, scarring, and extracellular matrix (ECM) remodeling [12,13,14,15]. A variety of cells in the skin and subcutis, present during wound healing and scarring, continuously sense a wide range of mechanical forces and consequently adapt to the new environment through shape modification, migration, proliferation, differentiation, and other biologic actions. The process through which cells perceive and respond to mechanical forces is called mechanotransduction. The mechanisms of mechanotransduction that modulate skin wound healing and scarring are not yet fully understood [16].

One of the most important employments of mechanotransduction is modulation of tissue stiffness. Matrix stiffness is an important micro-environmental cue that regulates cell behavior and function, the role of which has been proven in orienting cell division, maintaining tissue homeostasis, driving cell migration, and regulating differentiation [17,18]. In addition, in vivo tissues are viscoelastic and are made up of cells, extracellular fluid, and ECM. When ECM mechanics become imbalanced, pathological scarring may occur.

The above discussion suggests that mechanical effects on the body can have a therapeutic significance in a wide range of diseases, especially in pathological scarring. In clinical practice, combination therapies are needed to achieve a good scar outcome. Depending on the size, scar age, body location, origin of the scar, and the specific care demand of the patient, we have to adjust our scar treatment and choose the best option, which almost always consists of a combination of different treatments. This perspective article aims to translate research findings at the cellular and molecular levels into working mechanisms of physical anti-scarring interventions.

## 2. Mechanotransduction in Scarred Skin

It is important to grasp how mechanotransduction works at cellular and tissue levels. In scarring, mechanosensitive and mechanoresponsive cells recognize different external mechanical stimuli and this activates, suppresses, or modulates key molecules of the central mechanosignaling pathways [13]. Currently, scar mechanobiology research has resulted in the identification of a considerable number of these signaling pathways, mainly those in fibroblasts and myofibroblasts, which are often abundant in pathological scars and are the main effector cells of excessive extracellular matrix (ECM) deposition and contraction in scars [13].

Fibroblasts align and change their structure according to the direction of mechanical strain. Tension can alter the fibroblast expression of ECM remodeling and inflammatory genes [19]. High-tension forces on wounds are more likely to result in severe scarring. Tension-induced skin fibrogenesis is dependent on ECM cross-linking and stiffening and several mechanosignaling pathways involved in scarring, as there are: integrin-mediated transforming growth factor β (TGF-β) signaling, the integrin-FAK pathway, calcium-ion signaling, Wnt/β–catenin signaling, etc. [20].

The integrin-FAK pathway is the classical mechanotransduction pathway that regulates fibroblast viability, collagen production and reorientation, and fibroblast-to-myofibroblast differentiation. Integrins are likely the most important mechanical sensors and transfer information bidirectionally between the ECM and fibroblasts. Mechanical load is transferred from the cell surface through the ECM to cell-matrix adhesion sites located at the cell membrane (see Video animation S1). All matrix contacts contain integrin receptors that will act as mechanosensors. When bound to the ECM, these receptors become activated and their molecular shape is changed. Integrin heterodimers (e.g., alpha5beta1) bind to specific ECM components (e.g., fibronectin), and their cytoplasmic tails interact with adaptor proteins such as talin, vinculin, and paxillin that link them to the actin cytoskeleton (CSK). These proteins form a cytoskeletal complex, known as the focal adhesion complex (FAC), that physically links the integrins to the ends of contractile microfilament bundles (stress fibers), thereby forming a molecular bridge between the ECM and the CSK [21]. These FACs activate focal adhesion kinase (FAK), which plays a key early role in cell migration. FAK activity elicits intracellular signal transduction pathways that promote the turn-over of cell contacts with the ECM. When FAK is inhibited or knocked out, both pro-inflammatory and pro-fibrotic growth factors are downregulated, collagen deposition and myofibroblast amount are both reduced, and the mechanical force is uncoupled from fibrosis formation [22,23].

Another important fibrogenesis mechanism is the integrin-mediated TGF-β signaling. TGF-β is a soluble factor promoting fibrosis and is involved in myofibroblast differentiation [24]. TGF-β pathways are propagated downstream through the Smads pathway (a family of structurally similar proteins that are the main signal transducers for receptors of the TGF-B superfamily) and are associated with the Co-Smad to move into the nucleus, where they bind DNA and initiate target gene activation [25]. Moreover, TGF-β1 upregulates myofibroblast contractility, which is associated with pathological contractures, by inducing the formation of fibronectin fibrils. In the case of excessive scarring in the skin, normal healing fails, likely due to abnormal and excessive secretion of growth factors and/or a lack of molecules responsible for induction of apoptosis or remodeling of the ECM in normal healing [26].

## 3. Matrix Stiffness

Induced TGF-β1 expression and consequently fibroblast-to-myofibroblast differentiation are influenced by ECM stiffness. A stiff ECM sparks integrin to activate and release TGF-β1, the pro-fibrotic isoform of TGF-β. This leads to an increase in α-smooth muscle actin, which in turn increases ECM stiffness, again creating a positive feedback loop for fibrosis. Contrarily, soft ECM reduces the release of TGF-β1 and suppresses the expression of TGF-β1 and α-smooth muscle actin [21]. In healthy skin, the stiffness of the dermis is 1–5 kPa [27,28] (in terms of Young’s modulus), as opposed to fibrotic tissue and scars where the increase in stiffness is as high as 20–100 kPa. This is similar to the stiffness of tissue with a dense collagen structure, such as tendons [29]. However, the dermis in pathological scars builds up its stiffness gradually in the evolution as the initial provisional ECM in fresh wounds is reported to be very soft [27]. For therapeutic purposes, softening of the ECM stiffness can therefore be a potential target. This implies that mechanical force transmission is elevated in stiffer matrices and downregulated in softer matrices, leading to the hypothesis that physical scar management interventions should be aimed at softening of the ECM.

It is still difficult to draw a universal picture of the mechanical-based mechanisms in the complex micro-environment of scars in vivo. Next to the above-mentioned mechanisms, other mechanosignaling pathways are involved in the process of fibrosis through cell–cell interactions between fibroblasts, keratinocytes, endothelial cells, and adipocytes. These pathways are often induced by specific types of mechanical forces. Calcium-ion (Ca^2+^) signaling participates in integrin-dependent signaling and mediates actin-reorganization [30]. It is frequently induced by uniaxial stretching [31]. MAPK and G-protein signaling are closely interlinked with each other and are significantly activated by high-frequency repetitive stretching [32]. Wnt/β–catenin signaling is closely interlinked with TGF-β signaling and is reported to increase fibroblast proliferation, motility, and invasiveness [33]. Many recent papers also discuss the role of the HIPPO pathway, in which its two key downstream transcription coactivators, YAP and TAZ, are frequently reported to play an important role in dermal wound healing [34].

Since the role of these pathways in the working mechanisms of physical scar modalities is not substantiated by solid evidence, we limit ourselves to the integrin-mediated TGF-β signaling pathway and the integrin-FAK pathway.

## 4. Mechanomodulation of Matrix Stiffness

### 4.1. Physical Scar Management

Physical scar management is a collective name for all the non-invasive scar treatments that make use of physical interventions to act on the scar. Mechanical load is most interesting in this specific area of scar management and the development of effective treatments in scar after-care. We know that under the influence of mechanical tension, scar proliferation will be prolonged and will even prohibit a full maturation of the scar within the foreseen timeframe of wound healing of two years [15]. However, mechanical load is not just to be seen as a negative predictive factor in scar formation. Under controlled conditions, it can be used as a tool to regulate inflammation and fibrosis [15]. Khan et al. reclaimed the term “mechanotherapy” as “any intervention that introduces mechanical load with the goal of altering molecular pathways and inducing a cellular response that enhances tissue growth, modeling, remodeling, or repair” [35]. By understanding the mechanical stimuli to which connective tissue cells best respond and the mechanisms these cells use to convert mechanical signals into cellular responses, scar therapists may be able to modulate the response of physical interventions in scar treatment in order to obtain a beneficial effect on the remodeling of the ECM. Nearly every physical scar management introduces mechanical load (pressure garments, silicones, manual massage techniques, mobilizations, shock wave therapy, adhesive tape). However, the optimal duration, the frequency, and the intensity of the applied force to generate a beneficiary effect remain unclear.

### 4.2. Interactions between Extracellular Matrix and Cytoskeleton

**Figure 1:**
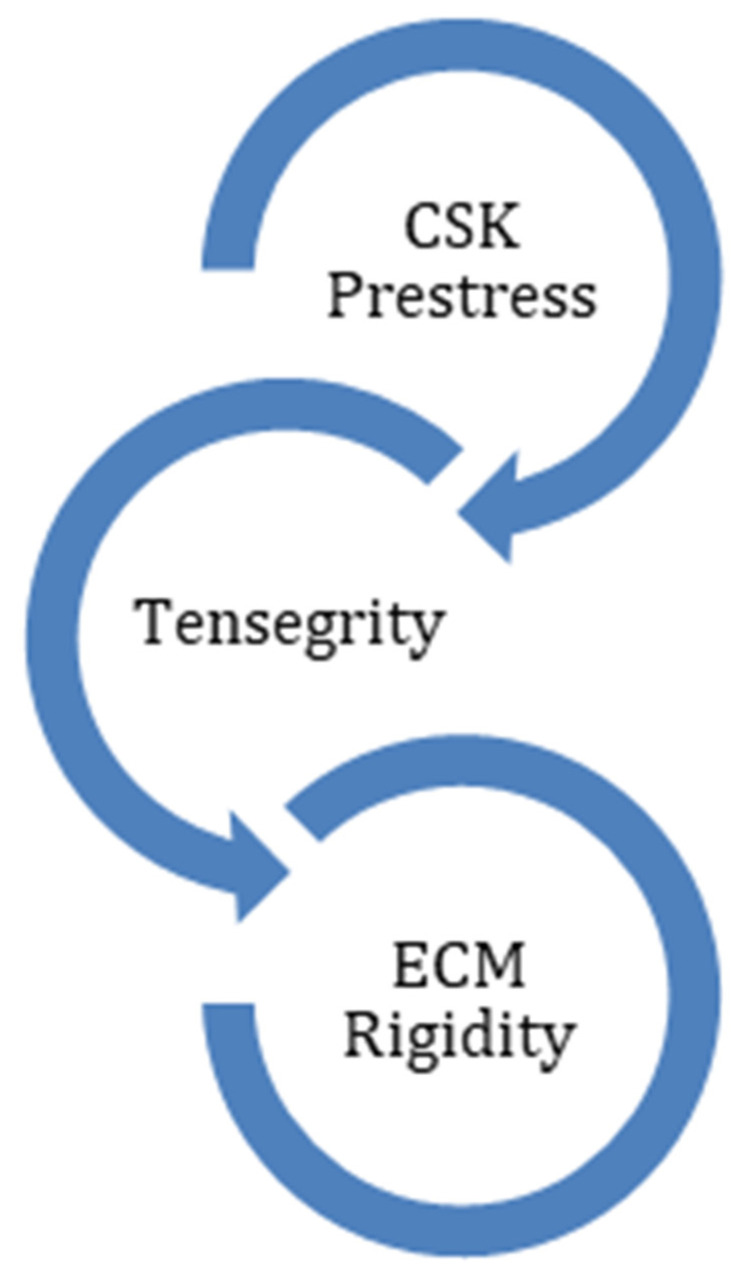
Tensegrity keeps the balance between CSK pre-stress and ECM rigidity.

An important characteristic of the cell in transferring the mechanical force between the ECM and the CSK is the continuous natural cellular pre-stress. Dermal cells use tensegrity (tensional integrity) to control their shape, structure, and pre-stress level (Figure 1). The concept of tensegrity was first introduced by Ingber [36]. It describes the crosstalk between the ECM and the cytoskeleton of the cell from a mechanotransduction point of view. The cytoskeleton consists of a filament and tubule network. Internal as well as external mechanical forces influence this network and will convert mechanical forces into biochemical signals. The cell responds to the stimuli by altering formation, growth, proliferation, differentiation, migration, and apoptosis of the cell. Tensegrity is an architectural structure and supports the physiologic balance between the ECM elements and the cytoskeleton. When this balance is distorted by trauma or infection, skin diseases such as fibrosis and pathological scarring occur.

The ECM is a dynamic, mobile, and multifunctional regulator of cellular behavior. The cells use the elasticity/rigidity of the ECM microenvironment to actively exert traction force on the ECM, which in turn alters the ECM [13].

When the external tension on the ECM increases, the CSK stiffness will also increase. This process is also known as “strain hardening”. This obviously implies an intense relationship between matrix rigidity and cell stiffness.

Fibrotic collagen networks can be locally aligned by cellular contraction, which results in a high degree of matrix stiffening. The alignment of collagen fibrils enables long-range force transmission to propagate contractile signals much further than soft matrices [37]. Another interesting fact is that inflammatory mediators during wound healing increase CSK pre-stress and thus render fibroblasts more sensitive to mechanical force [38].

All these findings lead to the conclusion that ECM rigidity and CSK pre-stress are interactively connected in the transmission of mechanical forces. In fibrotic connective tissue, the elevation of matrix stiffness, induced by pathological accumulation of ECM components and persistent inflammation, makes the fibroblast more sensitive to the mechanical force. This seems to indicate that lower loading rates than normal already initiate a response cascade, and that loading rates previously indicated as normal in healthy tissue induce pathological scarring. The aim of every physical intervention on young inflamed scars should therefore be to soften the ECM and CSK pre-stress by utilizing lower loading rates than normally used in healthy tissue (Table 1). Today, this proposed approach has already been implemented in clinical treatment modalities such as extracorporeal shockwave therapy [39,40,41] and serial casting [42]. The above-explained working mechanism of mechanosignaling pathways suggests that this hypothesis can be transferred to other physical treatment modalities (e.g., manual massage techniques, scar stretching, or vacuum massage) [43,44].

## 5. Dose Dependency of Applied Forces during Physical Scar Management

Different body parts exhibit different mechanical environments, from very dynamic such as in the heart, to an almost static environment in the skin. Therefore, besides the natural pre-stress in or realignment of the ECM and the CSK, feedback mechanisms that sense changes and restore values to normal are highly dependent on the magnitude, duration, and frequency of the applied mechanical load.

Cells react to cyclic strain by actively remodeling and reorienting the CSK, thus over longer periods they may differentiate to maintain their natural pre-stress [45]. The extent of cytoskeletal realignment can depend on the frequency and magnitude of the applied load [46], although realignment is absent in very compliant matrices [47]. Jungbauer et al. showed that there is no significant cell shape change in human dermal fibroblasts under the threshold of 2% applied strain [46]. Various studies have shown that mid-level strain magnitudes (10–20%) represent the most significant mechanotransduction effects [48,49,50].

Bouffard et al. reported significantly lower TGF-β1 protein levels and decreased type-I procollagen synthesis for tissues stretched over a brief time (10 min) with moderate amplitude (20%), when compared with non-stretched tissues [51], which also emphasizes the importance of the duration of the applied force.

**Figure 2:**
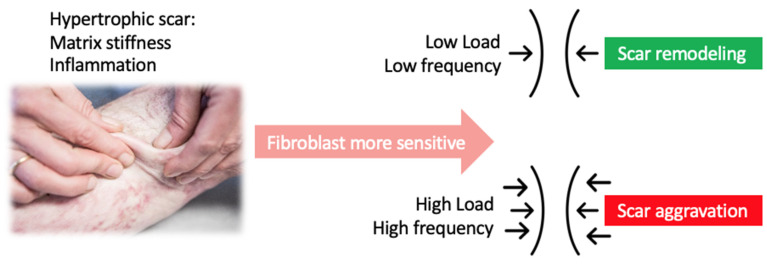
Moderate intensity and frequency of mechanical stimuli are indicated in physical scar management.

These results suggest that moderate amplitude, duration, and frequency are indicated in physical scar management (Figure 2). Future clinical studies should therefore compare physical therapeutic interventions with different intensities, application times, and pace.

## 6. Physical Modalities That Improve Tissue Stiffness

There is a wide variety of non-invasive treatment options that make use of mechanomodulation to improve various scar symptoms, including but not limited to manual scar massage, vacuum massage, pressure therapy, silicone therapy, hydrotherapy, scar taping, and shockwave therapy. We will sum up the evidence for the three treatments most frequently mentioned as mechanomodulation modalities to treat scars: silicone therapy, scar taping, and shockwave therapy. To further strengthen the clinical feasibility of these interventions in the future, cost-effectiveness studies are an important addition to the studies on the underlying mechanism and clinical outcome referred to in this paper.

### 6.1. Silicone Therapy

Silicone gel sheets (SGS) and silicone gel (SG) have been used to treat hypertrophic scars since the 1980s [52,53]. Although several studies have demonstrated the clinical efficacy of this therapy [54,55,56,57,58,59,60], its mechanism of action remains understudied. Mustoe [61] suggested that occlusion and hydration downregulate keratinocyte stimulation and in turn cause a decrease of fibroblast activity. Other studies demonstrated that SGS decreases TGF-β1 and TGF-β2 expression in fibroblasts [62,63], and Kikuchi and co-workers [64] demonstrated that scars treated with silicone gel show favorable gene expression for wound healing. The application of SG promoted the maturation of burn scars in most cases and significantly improved the surface roughness of the scars [65]. Statistically speaking, SGS is not different from SG when clinically assessed with the Vancouver Scar Scale [66].

Initially, the underlying working mechanisms of SGS were either not considered or linked to the effects of mechanical forces. Insight in mechanotherapy enlarged the possible concept of underlying working mechanisms. Akaishi et al. [67] provided evidence that SGS reduces tensile stress in the scar area, thus suggesting that SGS could be used as a mechanomodulation tool. SGS was effective in reducing the tension at the border between the scar and healthy skin. The SGS transferred the tension from the border of scars to the lateral edge of the SGS.

**Figure 3:**
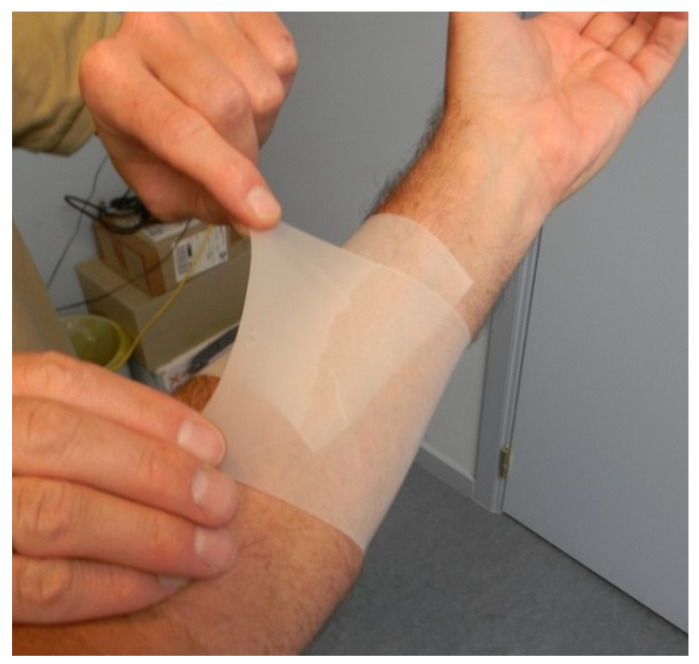
Gecko tape, a cohesive silicone bandage.

Van den Kerckhove et al. added another mechanotransduction force to the silicone application in terms of compression [68]. They described an inflatable silicone insert to treat scars (ISIS^®^) in which the pressure on the scar can be adjusted by means of a pump. This combination of pressure and silicone is still a point of discussion. Several studies showed controversial results in objectively improving outcomes such as scar thickness, itch, and redness, or improving scar scale ratings [69,70,71]. The authors of this paper on the other hand demonstrated that the addition of pressure to a silicone cohesive bandage (Figure 3) resulted in a significantly better scar elasticity when compared to SGS [72]. This study was the first to investigate the clinical effects of silicone combined with pressure as a mechanomodulation modality to improve scar stiffness.

### 6.2. Scar Taping

Based on the aforementioned increased mechanical tension hypothesis, it makes sense to minimize mechanical forces after surgery or spontaneous healing of (burn) wounds. Tension on a scar in one direction will result in a stretched scar. Multi-directional or cyclical tension on a scar will result in a hypertrophic scar [73,74]. Clinical experience has shown us that the most reliable way to support a scar is using tape, and initial evidence of mixed levels suggests some benefits of tapes for the management of hypertrophic scars and keloids [75,76]. Reiffel et al. [77] suggested longitudinal taping in the direction of the scar rather than at right angles to it. A strip of paper tape, approximately two to four inches longer than the wound, was applied to the normal skin, beyond the wound. The tape was then pulled as it was laid down, compressing it. It was usually changed daily and could develop blisters under the tape, although a number was not mentioned. Noncompliance of some patients was reported. Atkinson et al. [78] recommended multidirectional application of paper tape as scar support. Paper tape is rigid and can prevent an increase of tension but will not be able to reduce the already existing tension. They reported adverse reactions such as localized red rash (12%) and increased rate of wound infection (9%). A drop-out rate of more than 40% was noted and attributed to the possibility that the treatment demands were higher than the anticipated benefits. One randomized, double-blind trial with 195 patients using non-elastic adhesive tape applied perpendicular to the suture wounds of torso scars after dermatologic surgery reduced the scar width by 1 mm. When compared with normal dressings, skin taping on torso wounds for 12 weeks resulted in better scar appearance at 6 months [79]. None of these studies investigated whether the application of paper tape did actually reduce tension at the wound/scar site.

**Figure 4:**
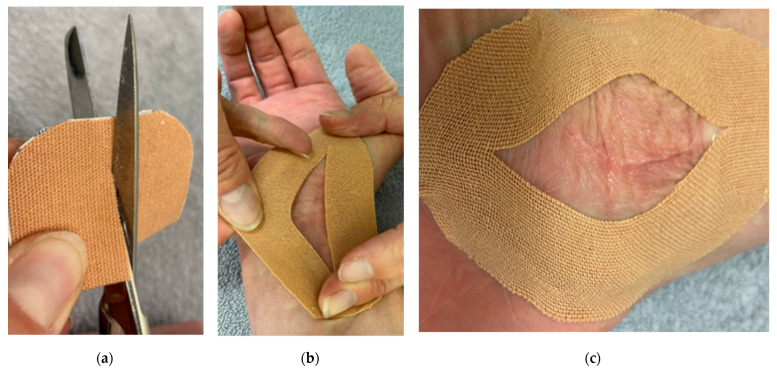
A new technique for tension-reducing tape application using elastic therapeutic tape. A longitudinal incision is made in the middle of the tape at approximately 5 mm from both ends (**a**). By means of this incision, the tape can be applied around the scar tissue, approximating the scarred skin from the ends towards the middle, horizontally as well as vertically (**b**). In this way, tension reduction can be obtained in all directions (**c**).

The authors of this manuscript developed a new technique for tension-reducing tape application using elastic therapeutic tape. It provides multidirectional tension relief. A longitudinal incision is made in the middle of the tape at approximately 5 mm from both ends (Figure 4a). By means of this incision, the tape can be applied around the scar tissue, approximating the scarred skin from the ends towards the middle, horizontally as well as vertically (Figure 4b). In this way, tension reduction can be obtained in all directions (Figure 4c), without touching the scarred skin, thus avoiding maceration of the wound/scar site and even allowing silicone application on the scarred skin. This technique also allows movement without increasing tension at the wound/scar site. A proof-of-concept study investigated whether this technique could actually reduce tension at the scar site. Elasticity was measured before application of the tape with a Cutometer^®^. This measurement was repeated after the application of the tape. The results of 20 patients showed a statistically significant difference in the scores for elasticity before and after tape application, corresponding to a reduction of tension in the middle of the scar site of 47%. This initial result indicates that the taping technique is actually reducing tension and can therefore decrease scar hypertrophy [80].

### 6.3. Shockwave Therapy

**Figure 5:**
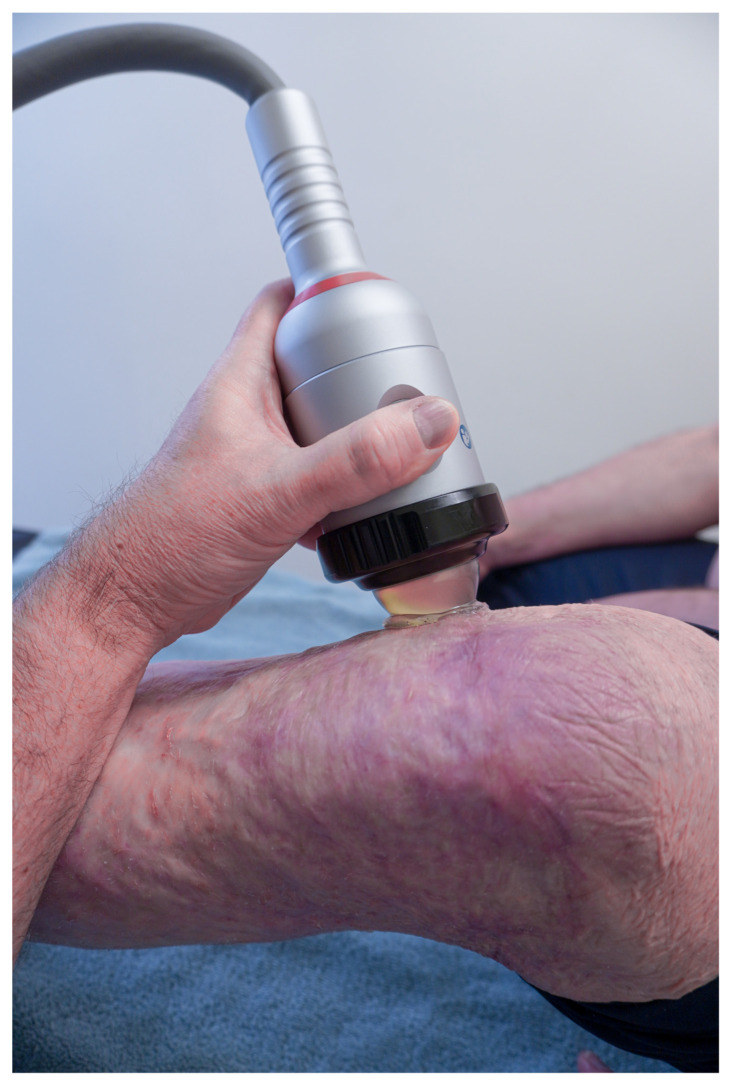
Shockwave application to treat scars.

The application of shockwave therapy in scar management (Figure 5) is still in its exploration phase, however there are some interesting findings. As in some other mechanotherapies applied in clinical practice, the main action of SWT seems to focus on inducing tissue regeneration and matrix remodeling “in vivo” by triggering mechano- and anti-inflammatory signaling pathways [81]. SWT is probably the most studied physical application to improve pathological scarring, in terms of basic as well as clinical research.

For a full treatment outline, the energy flux density (EFD), the number of pulses, the pulse frequency, and the number and interval of treatments are the most relevant parameters [82]. Differences in these parameters can lead to varying outcomes, emphasizing the dose dependency of these mechanotransduction events [81]. High-energy SWT can suppress cell growth, while lower-energy shock waves might enhance cell proliferation [41]. A study by Lee et al. [83] showed that the EFD plays an important role in the targeting of specific mechanosignaling pathways, with 0.12 mJ/mm^2^ being the optimal dose for activating the mTOR-FAK pathway and 0.10 mJ/mm^2^ showed the best results for inhibiting the TGF-β1/Smad pathway [84].

In the past two years, a high number of publications on the effects of SWT on burn scars was presented. A double-blinded, randomized, controlled trial of 48 patients with a burn to their dominant right hand revealed beneficial effects on pain, scar thickness, vascularity, and hand function [85]. Another study compared the efficacy of Triamcinolon Acetonide injections (TAI) alone, or in combination with SWT, to treat keloids. The SWT group showed significantly greater good to excellent improvements in the patient and observer global assessment [86]. Aguilera-Saëz et al. [87] found no significant differences in any of the Vancouver Scar Scale variables, comparing a group of burn patients treated with standard of care with a group additionally treated with SWT.

The authors of this paper carried out a prospective, randomized, placebo-controlled study on 40 patients with burn scars within 3 months of full wound closure [88]. Evaluations included the Patient and Observer Scar Assessment Scale (POSAS) for scar quality, tri-stimulus colorimetry for redness, tewametry for trans-epidermal water loss (TEWL), and cutometry for elasticity. Patients were randomly assigned to one of two groups, the low-energy intervention group or the placebo control group, and were tested at baseline, and after one, three, and six months. All patients were treated with pressure garments, silicone, and moisturizers. Both groups received the ESWT treatment (real or placebo) once a week for 10 weeks. The results showed a statistically significant better performance of the intervention group when assessing elasticity measured with cutometry. This finding leads to the hypothesis that SWT also has a beneficial influence on matrix stiffness by means of mechanotransduction effects [88].

## 7. Conclusions

The working mechanisms of physical anti-scarring modalities remained unclear for many years. Recent evidence underpinned the important role of mechanical forces in scar remodeling, especially the balance between matrix stiffness and cytoskeleton pre-stress. Mechanomodulation of scars applied with the right amplitude, frequency, and duration induces ECM remodeling and restores the ‘tensile’ homeostasis. Depending on the scar characteristics, specific (combinations of) non-invasive physical scar treatments are possible. Future translational studies are needed to define the dose dependency of mechanical interventions in human in vivo scar models through controlling mechanical load application and through measuring cellular and molecular responses in scar tissue by (immune)histological and molecular pathways’ analysis. The effect of the ECM rigidity and inflammatory mediators on the dose dependency of mechanotherapy are highly relevant to include in future research. In addition, these studies should include the association between cellular and molecular changes and clinically relevant changes of the scar.

## Figures and Tables

**Table 1:** Effect of internal and external forces linked to therapy goals and possible modulation in physical scar management.

Determining Factors in Physical Scar Management	Scar Facts	Therapy Goal	Modulation	Clinical Application
Internal Forces				
**ECM st** **iffness**	Fibrosis (alignment collagen) increases ECM stiffness [11,14].	Decrease ECM stiffness (and decrease fibroblast sensitivity).	Slow mechanical load rate -> decrease ECM stiffness [40,41,42,43,44,45,46]. Fast mechanical load rate -> increase ECM stiffness [40,41,42,43,44,45,46].	Vacuum massage, manual skin techniques
**CSK prestress**	Inflammation and ECM stiffness increases CSK prestress and fibroblast sensitivity [11,15].	Decrease CSK prestress.	Balance between internal and external forces -> induce CSK prestress [31]. Increased external forces -> increase CSK prestress [31].	Tape application
**External Forces**				
**Tensile force**	External forces at the epidermis are shear forces due to friction, tensile forces and compression forces. These forces will increase tension in the dermis.	Balance between internal and external forces.	Intensity/amplitude: <2% no effect [41] 10-20% significant effect [43,44,45] Frequency: low frequency, cyclic strain [40,43] Duration: moderate (no sustained signals) [46]	Vacuum massage, manual skinfold technique
**Compressive force**				Compressure garments, silicones, shockwave
**Shear force**				Manual gliding and splitting-up technique

## Supplementary Materials

The following supporting information can be downloaded at: https://www.mdpi.com/article/10.3390/ebj3020021/s1: Video animation S1: Journey of a mechanical stimulus from outside the skin to the cytoskeleton of a dermal cell.
